# Recent Evidence on Advances in PCI Treatment for Left Main Coronary Artery Disease

**DOI:** 10.31083/j.rcm2311370

**Published:** 2022-10-31

**Authors:** Xian Jin, Kaifan Niu, Chengxing Shen

**Affiliations:** ^1^Department of Cardiology, Shanghai Jiao Tong University Affiliated Sixth People's Hospital, 200235 Shanghai, China

**Keywords:** left main coronary artery disease, percutaneous coronary intervention, coronary artery bypass grafting

## Abstract

Revascularization therapy significantly improves the outcomes of patients with left main coronary artery disease (LMCAD), compared with medical therapy alone. For many years, coronary artery bypass grafting (CABG) has been the primary and standard treatment strategy. However, with advances in percutaneous coronary intervention (PCI) techniques and improvements in patients’ outcomes, there is growing evidence supporting PCI for LMCAD. In this review, we aim to integrate the available evidences on advances in PCI treatment for LMCAD and provide guidance for further clinical practice.

## 1. Introduction

Significant stenosis of the left main coronary artery (LMCA) is present in around 7% of patients undergoing coronary angiography [1]. Since approximately 2/3 of the cardiac blood flow is supplied by the left main artery, patients with significant left main coronary artery disease (LMCAD) often have poor prognosis [2]. Revascularization therapy greatly improves the outcomes of patients with left main disease, compared with medical therapy alone [3]. Traditionally, coronary artery bypass grafting (CABG) has been the primary treatment strategy, however, with advances in percutaneous coronary intervention (PCI) techniques and improvements in patients’ outcomes, more and more patients with LMCAD are receiving PCI, and there is growing evidence supporting PCI for LMCAD. In this review, we aim to integrate the available evidence on advances in PCI treatment for left main coronary artery disease and provide guidance for further clinical practice.

## 2. PCI or CABG

There has been controversy for many years over whether PCI in the era of drug-eluting stents (DESs) can replace or even be superior to CABG in treating patients with LMCAD.

The latest clinical findings in recent years, especially the 5-year follow-up results of the four most representative large randomized controlled trials (RCT), including SYNTAX, PRECOMBAT, NOBLE, and EXCEL, have brought more conclusive evidence and deeper understanding on how to choose the best revascularization strategy for LMCAD. The detailed 5-year follow-up results from these RCTs are shown in Table 1. The 5-year follow-up results from the SYNTAX (Synergy Between PCI With Taxus and Cardiac Surgery) trial [4] which enrolled 705 patients with LMCAD (PCI 357 vs. CABG 348) showed that no significant difference was noted between PCI and CABG in overall major adverse cardiac and cerebrovascular events (MACCEs) (36.9% vs. 31.0%, *p* = 0.12) nor in mortality (12.8% vs. 14.6%, *p* = 0.53). A lower incidence of stroke (1.5% vs. 4.3%, *p* = 0.03) was seen in patients who received PCI instead of CABG, while a significant increase of repeat revascularization was observed in the PCI group (26.7% vs. 15.5%, *p *< 0.01). The PRECOMBAT (Premier of Randomized Comparison of Bypass Surgery versus Angioplasty Using Sirolimus-Eluting Stent in Patients with Left Main Coronary Artery Disease) trial [5] included 600 patients (PCI 300 vs. CABG 300) and showed no difference in overall MACCEs rate (PCI 17.5% vs. CABG 14.3%, *p* = 0.26) nor in composite endpoints including all-cause mortality, myocardial infarction (MI) and stroke (PCI 8.4% vs. CABG 9.6%, *p* = 0.66) between the two groups, while ischemia-driven target vessel revascularization (TVR) was significantly higher after PCI (11.4% vs. 5.5%, *p* = 0.012). The EXCEL (Evaluation of XIENCE versus Coronary Artery Bypass Surgery for Effectiveness of Left Main Revascularization) trial [6], enrolling 1905 patients (PCI 948 vs. CABG 957) with LMCAD, is the largest randomized controlled trial to date comparing the two treatment strategies of PCI and CABG. In five years, no significant difference in primary outcome events combing all-cause mortality, stroke, and myocardial infarction was discovered between the two treatment strategies (PCI 22.0% vs. CABG 19.2%, *p* = 0.13). However, it should be noted that patients in the PCI group exhibited higher incidence of all-cause death (13.0% vs. 9.9%, *p *< 0.05) but no significant increase of cardiac death (5.0% vs. 4.5%), while patients in the CABG group experienced higher rate of stroke events (2.9% vs. 3.7%, *p *> 0.05). It was also noted that ischemia-driven repeat revascularization was evidently higher in patients undergone PCI compared with those in the CABG group (16.9% vs. 10%, *p *< 0.001). There were 1201 patients (PCI 598 vs. CABG 603) participated in the NOBLE (Nordic-Baltic-British Left Main Revascularization) trial [7] and no significance was found in all-cause mortality between the two groups (PCI 9% vs. CABG 9%, *p* = 0.68). The rate of non-procedural myocardial infarction (8% vs. 3%, *p* = 0.0002) and repeat revascularization (17% vs. 10%, *p* = 0.0009) was higher in the PCI group, and the incidence of MACCEs was 28% after PCI compared to 19% after CABG (HR 1.58 [95% CI 1.24–2.01], *p* = 0.0002) with a hazard ratio exceeding the limit for non-inferiority test. The NOBEL trial with a non-inferiority design was initially intended to explore whether PCI could replace CABG, however, the results showed that CABG was significantly superior to PCI in treating LMCAD.

**Table 1.** **Overview of 5-year follow-up results from current randomized controlled trials comparing PCI versus CABG for patients with LMCAD**.

	Primary outcomes at 5-year follow-up (PCI vs. CABG)			
Study	SYNTAX	PRECOMBAT	EXCEL	NOBLE
No. of patients	705	600	1905	1184
	357 vs. 348	300 vs. 300	948 vs. 957	598 vs. 603
SYNTAX score (mean)	30	25	20	22
Overall MACCEs	36.9% vs. 31.0%	17.5% vs. 14.3%	-	28% vs. 19%*
All death/stroke/MI	19.0% vs. 20.8%	8.4% vs. 9.6%	22.0% vs. 19.2%	-
All-cause mortality	12.8% vs. 14.6%	5.7% vs. 7.9%	13.0% vs. 9.9%*	9% vs. 9%
Cardiac death	8.6% vs. 7.2%	3.8% vs. 6.9%	5.0% vs. 4.5%	4% vs. 4%
Stroke	1.5% vs. 4.3%*	0.7% vs. 0.7%	2.9% vs. 3.7%	4% vs. 2%
Myocardial infarction	8.2% vs. 4.8%	2.0% vs. 1.7%	10.6% vs. 9.1%	8% vs. 3%*
Total repeat revascularization	26.7% vs. 15.5%*	13.0% vs. 7.3%*	17.2% vs. 10.5%*	17% vs. 10%*

*p < 0.05. Percentages are Kaplan-Meier estimates from the intention-to-treat analysis with log-rank *p* values calculated. PCI, percutaneous coronary intervention; CABG, coronary artery bypass grafting; MACCE, major adverse cardiovascular and cerebrovascular event; MI, myocardial infarction.

The SYNTAX score is an angiographic assessment of the coronary vasculature and reflects complexity of coronary artery disease, with scores ≤22 defined as low, 23–32 as intermediate, and ≥33 as high in accord with disease complexity. Of note, the mean SYNTAX score of LMCAD enrolled in these four RCTs was 25, and the majority of patients had low (41%) or intermediate (37%) coronary anatomical complexity. All patients in the above-mentioned trials undergone PCI were implanted with DESs, and only 22% of patients had a SYNTAX score ≥33 which is considered high complexity LMCAD, so it can relatively objectively reflect the efficacy of contemporary PCI compared with CABG. Furthermore, second-generation DESs were applied in the NOBEL and EXCEL trials which is in accord with the latest PCI techniques, hence their results can reflect the difference in efficacy of current PCI and CABG treatment for LMCAD more realistically and offer strong guidance for clinical practice. Interestingly, although NOBEL and EXCEL have many similarities in study design as they enrolled patients with similar baseline characteristics, were completed at the same time, and both applied new generation DESs, the results of the two trials were quite different, with NOBEL indicating CABG being superior to PCI while EXCEL showing that PCI is not inferior to CABG. Due to the fact that MI in the EXCEL trial was defined as peri-procedural MI rather than the universal definition, which may lead to slightly higher incidence of MI in the CABG group, the comparison between EXCEL and other trials regarding the risk of MI might not be entirely accurate [8]. An individual patient data meta-analysis of these four clinical trials by Sabatine *et al*. [9] showed that there was no statistically significant difference in 5-year all-cause mortality between PCI and CABG in LMCAD patients with low or moderate coronary anatomical complexity (11.2% vs. 10.2%, *p* = 0.33), although Bayesian analysis suggested a possible advantage of CABG over PCI (more likely than not <0–2% per year). The incidence of MI (6.2%, vs. 2.6%, *p *< 0.0001) and repeat revascularization (18.3% vs. 10.7%, *p *< 0.0001) were higher in the PCI group. The lower risk of stroke after PCI occurred only in the first year after the procedure, while there was no difference in the risk of stroke on the whole between the two groups (2.7% vs. 3.1%, *p* = 0.36).

In recent years, 10-year follow-up studies on PCI and CABG have also been released, revealing a longer-term comparison between efficacy of the two treatment strategies. The detailed 10-year follow-up results are shown in Table 2. The PRECOMBAT 10-year follow-up data [10] showed no significant difference in death of any cause nor composite endpoint events including death, MI, and stroke in ten years between the two groups, while ischemia-driven target vessel revascularization was higher after PCI by 2-fold (16.1% vs. 8.0%). However, due to the unexpectedly low event rates of the PRECOMBAT trial, the study was underpowered. Thus, the lack of yearly clinical follow-up between 5 and 10 years after 5 years of follow-up may have led to an underestimation of clinical event rates. The LE MANS (Left Main Coronary Artery Stenting) trial [11] also showed no difference between PCI and CABG in terms of mortality rate and MACCEs rate, but the study was extremely limited as it only included 105 patients. The MAIN-COMPARE (Revascularization for Unprotected Left Main Coronary Artery Stenosis: Comparison of Percutaneous Coronary Angioplasty Versus Surgical Revascularization) observational registry [12] enrolled 2240 patients from the real world and showed that the composite endpoints such as death, MI and stroke were similar in both groups, but repeat TVR was significantly higher in the PCI group. Finally, in 10-year follow-up data from the SYNTAX extended survival study [13], no statistically significant difference was discovered in mortality rate between PCI and CABG. As in the above-mentioned follow-up studies, the stents used for PCI were former bare-metal stents (BMSs) or first-generation DESs, but the long-term comparison revealed that PCI was not inferior to CABG in terms of mortality.

**Table 2.** **Overview of 10-year follow-up results from current randomized controlled trials comparing PCI versus CABG for patients with LMCAD**.

	Primary outcomes at 10-year follow-up (PCI vs. CABG)			
Study	SYNTAXES	PRECOMBAT	LE MANS	MAIN-COMPARE
No. of patients	1800	600	105	2240
	903 vs. 897	300 vs. 300	52 vs. 53	1102 vs. 1138
SYNTAX score (mean)	29	25	25	-
Overall MACCEs	-	29.8% vs. 24.7%	51.1% vs. 64.4%	-
All death/stroke/MI	-	18.2% vs. 17.5%	-	25.0% vs. 24.6%
All-cause mortality	27% vs. 24%	14.5% vs. 13.8%	21.6% vs. 30.2%	22.2% vs. 21.4%
Cardiac death	-	7.8% vs. 8.7%	-	-
Stroke	-	1.9% vs. 2.2%	4.3 vs. 6.3%	-
Myocardial infarction	-	3.2% vs. 2.8%	8.7 vs. 10.4%	-
Total repeat revascularization	-	21.3% vs. 10.6%*	26.1% vs. 31.3%	22.6% vs. 5.4%*

*p < 0.05. Percentages are Kaplan-Meier estimates from the intention-to-treat analysis with log-rank *p* values calculated. PCI, percutaneous coronary intervention; CABG, coronary artery bypass grafting; MACCE, major adverse cardiovascular and cerebrovascular event; MI, myocardial infarction.

Some other meta-analyses [14, 15] showed that in low and intermediate complexity of LMCAD, risk of all-cause death and cardiac death after PCI were comparable to that after CABG, while risk of repeat revascularization was significantly higher after PCI.

Although there is not sufficient evidence to date that PCI can be a perfect substitute for CABG, an increasing number of studies have proved that with advances in PCI techniques [4, 5, 6, 7, 10, 11, 12, 13], the efficacy of PCI is approaching or even comparable to that of CABG regarding all-cause mortality and cardiac death for patients with low and moderate complexity of LMCAD, however, it is undeniable that ischemia-driven revascularization is still higher with PCI. Currently, PCI and CABG should be complementary treatment strategies for stable LMCAD, and how to choose the best treatment strategy should depend on a full evaluation of many factors including anatomy of the coronary artery, the SYNTAX score, overall medical conditions, and preference of the patient.

## 3. Ostial/Midshaft and Distal LMCAD

Left main coronary artery disease can be classified into ostial, midshaft, and distal lesions according to location of the stenosis. Compared with distal bifurcation LMCAD, ostial/midshaft lesions are often regarded less challenging with more favorable outcomes, and the efficacy of PCI and CABG were comparable in terms of ostial/midshaft lesions in 5-year follow-up study [16]. The 12-year follow-up results of another observational study [17] also showed that the long-term outcomes of PCI and CABG were generally comparable for ostial/midshaft left main lesions. In addition, Yoon *et al*. [18] analyzed 2112 cases with ostial/midshaft LMCAD from the IRIS-MAIN (Interventional Research Incorporation Society-Left MAIN Revascularization) registry study (PCI 1329 vs. CABG 783) and stratified the patients by stent type availability as BMSs, first-generation DESs and second generation-DESs. The results revealed that with iterations of stenting techniques, the efficacy of PCI treatment for LMCAD improved greatly, approaching the efficacy of CABG. However, the distal bifurcation lesions are more commonly seen in patients with LMCAD, accounting for approximately 80% of all left main lesions [19], the prognosis of which is worse than ostial/midshaft lesions due to the involvement of bifurcation and complicated anatomy [20, 21]. The 12-year follow-up results of the MAIN-COMPARE trial [22] found that there was no difference in the rate of composite endpoint events and mortality between PCI and CABG in ostial/midshaft lesions, while the efficacy of PCI was significantly inferior to that of CABG in terms of distal LMCAD. A subgroup analysis of the EXCEL trial [23] showed that, as for low and moderate complexity of LMCAD with SYNTAX scores below 32, the composite endpoint events defined as death, MI and stroke at 3 years of follow-up were comparable after PCI and CABG with both distal LMCAD (PCI 15.6% vs. CABG 14.9%, *p* = 0.61) as well as ostial/midshaft LMCAD (PCI 12.4% vs. CABG 13.5%, *p* = 0.77), and no difference in ischemia-driven repeat revascularization was seen with ostial/midshaft lesions between the two treatment strategies (PCI 9.7% vs. CABG 8.4%, *p* = 0.68), while repeat revascularization with distal bifurcation lesions after PCI was significantly higher than that after CABG (13.0% vs. 7.2%, *p* = 0.0001).

As a result, the current available evidence suggests that the overall efficacy of PCI for ostial/midshaft LMCAD is close to or even comparable to that of the standard treatment CABG, whereas PCI for distal LMCAD is still slightly inferior to CABG, with the main weakness being the higher rate of ischemia-driven repeat revascularization. When evaluating and considering PCI as the treatment strategy for LMCAD, the 2018 ESC/EACTS Guidelines [24] suggested based on available evidence that PCI can be considered for patients with low to moderate SYNTAX scores (0–22, level of evidence: I; 22–32, level of evidence: IIa) regardless of the location of the diseased LMCA, whereas the AHA/ACC guidelines [25] gave different recommendations of PCI for LMCAD according to the location of the lesions, namely a class IIa recommendation for ostial/midshaft LMCAD and a class IIb recommendation for distal LMCAD (level of evidence: IIb).

## 4. Single-Stenting or Dual-Stenting

When treating LMCAD with PCI, the first decision to make is whether to propose a single-stenting or dual-stenting technique. According to current consensus, a provisional single stent is the preferred strategy for LMCAD without significant lesions at the side branch ostium [26]. A secondary analysis of the EXCEL trial [27] showed that the provisional strategy was better than upfront dual-stenting for LMCAD without true distal bifurcation lesions in reducing the composite endpoint events including death, MI and stroke at 3 years of follow-up (13.8% vs. 23.3%, *p* = 0.03). In the case of true distal left main bifurcation diseases, the 3-year follow-up results of the DKCRUSH-V trial (Double Kissing Crush versus Provisional Stenting for Left Main Distal Bifurcation Lesions: The DKCRUSH-V Randomized Trial) [28] revealed that double-kissing crush (DK-Crush) dual-stenting was superior to the provisional strategy in composite endpoints defined as target lesion revascularization (TLR), target vessel myocardial infarction (TV-MI) and cardiac death (8.3% vs. 16.9%, *p *< 0.01). In contrast, the EBC MAIN (European Bifurcation Club Left Main Coronary Stent Study) [29] comparing a stepwise provisional strategy with a systematic dual-stent approach in distal LMCAD affecting both ostia of left anterior descending (LAD) and left circumflex (LCX) arteries showed that the two treatment strategies had comparable effects in terms of combined endpoints including all-cause death, MI and TLR at one year (14.7% in the single-stent group vs. 17.7% in the dual-stent group, *p* = 0.34).

The distinguished results of the two studies [28, 29] might be due to the following reasons. Firstly, the lesions included in the DKCRUSH-V trial were more complex with a mean SYNTAX score of 31 and side branch lesions >10 mm in length with a mean of 16 mm, compared to a mean SYNTAX score of 23 and side branch lesions <10 mm with a mean length of 7 mm in the EBC MAIN study. The primary endpoint of DKCRUSH-V is more concerned with the difference in stent-related events such as cardiac death, TV-MI and TLR, whereas the EBC MAIN adopted a more patient-oriented primary endpoint including all-cause death, any MI and TLR. Furthermore, the patients enrolled in the EBC MAIN were older (70 vs. 64) with more comorbidities.

In addition, the recent DEFINITION (Definitions and impact of complEx biFurcation lesIons on clinical outcomes after percutaNeous coronary IntervenTIOn using drug-eluting steNts) II study [30] with similar side branch lesion enrollment criteria as DKCRUSH-V including LCX stenosis severity ≥70% and extension of the lesions ≥10 mm showed that the rates of TV-MI and TLR were dropped after the application of the upfront dual-stent strategy, as compared with the provisional approach. For stenosis of side branch ostia <70% and length of lesions <10 mm, no significant difference was observed in terms of target lesion failure (TLF) between the provisional and the DK-Crush strategies in the DKCRUSH-V trial [31].

These findings suggest that, for non-true distal bifurcation LMCA lesions, the provisional single stenting is the preferred standard treatment strategy, and for true distal bifurcation lesions, efficacy of provisional stenting and upfront dual-stenting are equivalent, whereas for complex true bifurcation lesions with side branch ostia stenosis >70% and lesion length >10 mm, dual-stent strategy may be superior to the provisional strategy.

## 5. Dual-Stenting Techniques

Currently, the most commonly used dual-stenting techniques are T-stent, T and protrusion (TAP), Culotte, and DK-Crush. It is important to note that T-stent, TAP, and Culotte techniques can be used in a provisional strategy in a bailout indication such as dissection and flow compromise *etc*. as well as in a planned dual-stent strategy. The BBK (Bifurcations Bad Krozingen) II study [32] showed that the Culotte technique was able to reduce risk of restenosis (6.5 vs. 17%, *p* = 0.006) and target lesion failure (6.7% vs. 12.0%, *p* = 0.069) compared to TAP. The DEFINITION III study [33] revealed that the DK-Crush technique was superior to the Culotte technique, reducing rate of TVR (4.3% vs. 11.0%, *p *< 0.05) and incidence of in-stent restenosis (ISR) in LCX (6.8% vs. 12.6%, *p *< 0.05) at one year. The advantage of DK-Crush over Culotte became more significant in case of side branch bifurcation angel ≥${\mathrm{70}}^{\circ }$ and high complexity lesions with SYNTAX score ≥23. In addition, a significant increase in MACCEs with the Culotte procedure (Culotte 23.7% vs. DK-Crush 8.2%, *p *< 0.001) was seen from the 3-year follow-up results of DEFINITION III study [34]. It should be noted that dual-stenting techniques are relatively complex and requires a high level of proficiency from the operators. Although there is no specifically preferred dual-stenting technique, both patients’ conditions and operators’ proficiency should be considered while choosing appropriate dual-stenting techniques for LMCAD.

## 6. POT and KBI

The proximal optimization technique (POT) is a mandatory step for unprotected left main coronary artery lesions regardless of true bifurcation lesions. The POT involves post dilation with a short balloon larger than the stent size positioning with its distal end in front of the carina, thus adapting the stent frame to the bifurcation anatomical morphology and correcting the proximal segment malapposition or underexpansion. A recent clinical registry study [35] enrolling 4395 LMCAD patients with bifurcation lesions supported the application of POT to reduce the incidence of TLF in true bifurcation lesion after PCI. Another commonly used technique for bifurcation lesions is kissing balloon inflation (KBI), which is a standard step when using a dual-stenting technique, but whether it is necessary as a routine procedure in a single-stent strategy remains controversial. The COBIS (Korean Coronary Bifurcation Stenting) II registry study [36] showed that final KBI in single-stent approach is associated with favorable long-term clinical outcomes and reduced TLR. The recent AOI-LMCA (Assessing Optimal Percutaneous Coronary Intervention for LMCA) study [37], on the other hand, indicated that performing final KBI did not affect TLR and other clinical outcomes at 5 years of follow-up. In the recent EXCEL study, the 4-year composite end points such as death, MI and stroke were not significantly different with or without final KBI for either single or dual stenting. The result that there was no evident advantage of final KBI for dual-stenting was quite unexpected, since final KBI was regarded essential for dual-stenting techniques with much clinical evidence to support it [38]. The recent RAIN-CARDIOGROUP VII study (Very Thin Stents for Patients With MAIN or BiF in Real Life: The RAIN, a Multicenter Study) [39] revealed that routine final KBI with dual-stenting reduced TVR and restenosis, and balloon with short proximal overlap contributed to better outcomes. However, the impact of the interaction between POT and KBI on procedural outcomes is still not clearly elucidated, and future studies are necessary to further clarify the role of the interaction between the two on procedural outcomes.

## 7. TRA or TFA

Transfemoral access (TFA) has been the traditional approach for PCI as it can provide easy access for catheters, stents or other devices through its larger caliber [40]. Over the years, more and more evidence has supported the transradial access (TRA) owing to less bleeding, fewer vascular complications, and better patients experience following TRA [40, 41]. As there are limited studies reporting the access types used in PCI for LMCAD, along with the fact that LMCAD is often more complicated and associated with worse prognosis, a number of professionals still preferred TFA over TRA since they are more experienced with TFA and can better handle emergent situations during the procedure. A cohort study including 931 patients from the EXCEL trial [42], with 248 (26.6%) patients undergoing TRA, have revealed that TRA and TFA are comparable in the risk of all-cause death, MI, TVR and stent thrombosis. A recent meta-analysis involving 12 observational studies [43] have indicated that, compared with TFA, TRA is associated with fewer bleeding outcomes and lower in-hospital mortality. Therefore, for the majority of patients with LMCAD, TRA is at least as safe as TFA, however, for patients with true distal bifurcations and complex anatomical characteristics, TFA might be the more appropriate approach.

## 8. Intracoronary Imaging Guidance

Intracoronary imaging techniques such as intravascular ultrasound (IVUS) and optical coherence tomography (OCT) during PCI can provide assessment and guidance that is essential to achieve good long-term outcomes for LMCAD. Intracoronary imaging can quantify lesion severity, guide lesion preparation with morphologic data, select appropriate stent size by accurate vessel sizing, identify the landing zone, diagnose acute vessel complication and define procedural success. Studies have proved that IVUS-guided PCI for LMCAD improves patient outcomes [44, 45, 46, 47]. OCT has a shorter wavelength and can provide higher resolution images (10–20 μm) than IVUS (150–200 μm), whereas the tissue penetration ability of OCT is weaker than that of IVUS. Therefore, the limitations of OCT may be more evident when imaging left main lesions. There may be difficulties in visualizing left main lesions >4 mm due to the lower field depth of OCT, and it is also relatively difficult to achieve a blood-free field within the LMCA. Despite these limitations, OCT measurements and evaluations are proven to be more accurate in the most common and complex left main distal bifurcation lesions [48]. Of note, the left main minimal luminal area (MLA) of OCT measurements are typically 10% smaller than that measured by IVUS [49]. The LEMON (LEft Main Oct-guided iNterventions) study [50] revealed that OCT-derived information including stent expansion, apposition and dissection at stent edges led to 26% altered procedural strategy, indicating the importance of OCT in guiding PCI for LMCAD. The ongoing OCTOBER (Optical Coherence Tomography Optimized Bifurcation Event Reduction) trial [51] intends to evaluate the effect of high resolution OCT in imaging stent implantation process of LMCA bifurcation lesions to guide important PCI procedural steps such as evaluation of plaque preparation and wire positions.

## 9. Anti-Platelet Strategy

Current practice guideline recommends applying dual anti-platelet therapy (DAPT), the standard anti-platelet strategy consisting of aspirin and oral P2Y12 inhibitors, for at least 12 months after PCI with DES for ACS patients [52]. For patients with LMCAD, there lacks sufficient data and universal consensus on DAPT duration after PCI in order to reduce the risk of ischemic events while minimize bleeding. Results of an observational study including 1827 patients who received PCI with DES for LMCAD from two large multicenter registries IRIS-MAIN and KOMATE (Korean Multicenter Angioplasty Team Study) [53] revealed that DAPT <12 months was in relation to significantly higher risk of MACCE while not associated with evidently reduced bleeding, compared to DAPT for 12–24 months. The DAPT score and PRECISE-DAPT (PREdicting bleeding Complications In patients undergoing Stent implantation and subsEquent Dual Anti Platelet Therapy) score designed to evaluate the benefits and risks of different DAPT durations [52, 54] can be used to identify patients with high risk of bleeding and guide individual DAPT duration strategy.

## 10. PCI Volume

To date, several studies have analyzed the effect of operators’ experience or PCI volume on procedural outcomes. Xu *et al*. [55] analyzed 1948 unprotected left main (uLM) PCIs performed by 25 operators and showed that although experienced operators performed procedures with more complexity, the 3-year follow-up results still showed that patients undergone procedures performed by experienced operators (≥15 uLM-PCI cases/year for 3 consecutive years) had better prognosis both in near and long term. Another study [56] involving 6724 uLM-PCI cases found significantly better 12-month survival (0.54 [95% CI, 0.39–0.73]; *p *< 0.001) in patients operated by operators with higher annual PCI volume (a mean of 21 procedures/year) compared to those with lower annual volume (a mean of 2 procedures/year), and suggested that the minimum individual uLM-PCI volume threshold associated with improved survival was ≥16 cases/year. In addition, the J-PCI registry (National PCI Data Registry) study [57] enrolling 24,320 PCI cases for unprotected LMCAD analyzed the effect of uLM-PCI outcomes based on the volume of the interventional center rather than the individual procedure volume, and showed that uLM-PCI outcomes were significantly better in large cardiac centers (annual PCI volume of 488–3015 cases) than in lower volume centers (annual PCI volume below 216 cases). Given that PCI for left main lesions is a relatively complex procedure, especially for distal bifurcation lesions, operators’ experience may be critical to the success of the procedure. Current guidelines [24] recommend that PCI for LMCAD be performed by operators with experience in 25 uLM-PCI cases per year.

## 11. Future Perspectives

Upgraded intracoronary imaging techniques and coronary functional assessment methods integrating hemodynamic and anatomical assessment of coronary artery will be more prevalent in the future, which can contribute to precise preoperative planning, timely intraoperative guidance and ambulatory monitoring of postoperative recovery. The innovation of stents (including the next generation of DES and bioresorbable scaffold) as well as drug-coated balloon (DCB) may further improve the efficacy, safety and long-term outcomes after PCI for LMCAD.

## 12. Conclusions

As devices and techniques advance rapidly, more patients with unprotected left main coronary artery disease are undergoing PCI, and in low and moderate complexity lesions with SYNTAX score of 32 or less, the long-term outcomes after PCI are comparable to those after CABG, although ischemia-driven repeat revascularization is still higher after PCI. For those intended to treat with PCI, whether to perform a provisional single-stent or a planned dual-stent strategy needs to be evaluated. Current available evidence supports the choice of a single-stent strategy through transradial access in most cases, that is, the simpler, the better. For complex distal true bifurcation lesions, an upfront dual-stent strategy with transfemoral access may be required, and the DK-Crush may be the preferred procedure for experienced operators. The guidance of intracoronary imaging has been proven to help improve the outcomes of PCI. Duration of dual anti-platelet therapy should be decided after full clinical evaluation for individual patients and is recommended to be no less than 12 months. Unprotected left main percutaneous coronary intervention is a relatively complex procedure and is recommended to be performed in large centers by experienced operators. In the future, with the help of upgraded intracoronary imaging techniques and overall functional assessment, along with application of new generation stents, PCI for LMCAD will be more precise with advanced efficacy and better prognosis in long term.
